# LapAppendectomy4all: validation of a new methodology for laparoscopic appendectomy simulation and training

**DOI:** 10.1007/s13304-025-02127-y

**Published:** 2025-02-08

**Authors:** Mário Rui Gonçalves, Ricardo Marinho, Sofia Gaspar Reis, Ricardo Viveiros, Manuel Moutinho Teixeira, Ana Kam Andrade, Maria do Carmo Girão, Pedro Pina-Vaz Rodrigues, Miguel Castelo-Branco Sousa

**Affiliations:** 1 Centro Académico Clínico das Beiras (Academic Clinical Center of Beiras), Faculty of Health Sciences, Faculty of Health Sciences, University of Beira Interior, Av. Infante D. Henrique, 6200-506 Covilhã, Portugal; 2 Faculty of Health Sciences, University of Beira Interior, Av. Infante D. Henrique, 6200-506 Covilhã, Portugal; 3 Centro Hospitalar Barreiro Montijo, Avenida Movimento das Forças Armadas, 2830-003 Barreiro, Portugal; 4 Hospital Central do Funchal, Avenida Luís de Camões nº 57, 9004-514 Funchal, Portugal; 5 Serviço de Cirurgia Geral, Unidade Local de Saúde de Castelo Branco, Hospital Amato Lusitano, Av. Pedro Alvares Cabral 3, 6000-085 Castelo Branco, Portugal; 6 Serviço de Cirurgia Geral, Unidade Local de Saúde de São José, Lisbon, Portugal; 7 Serviço de Cirurgia Geral, Unidade Local de Saúde do Baixo Alentejo, Beja, Portugal; 8 Centro Académico Clínico das Beiras (Academic Clinical Center of Beiras), Faculty of Health Sciences, University of Beira Interior, Av. Infante D. Henrique, 6200-506 Covilhã, Portugal

**Keywords:** Simulation, Education, Training, Simulation model, Appendectomy, Residency

## Abstract

Appendectomy, whether open or minimally invasive (MIS) is one of the most frequent procedures performed by young residents. We designed and tested a new methodology and a new inexpensive silicone model for Laparoscopic Appendectomy (LA) simulation. This study aimed to assess their fidelity, usefulness and educational value in an introduction to laparoscopy course. The course was open to first-year general surgery residents. The group was divided in two and one of the groups watched a video of the procedure before the simulation. Individual performances were assessed directly on the models, using a specific assessment scale. Participants answered a questionnaire at the end of the course for evaluation. Thirty-five residents participated in this study. Execution, quality, and global performance were higher in the group that had more experience with the model. Thirty-two trainees (91%) answered the questionnaire. There was a strong agreement that the model was adequate for this type of course and face and content validity was considered high/very high. Participants strongly agreed that this model gives more confidence to perform a real LA and almost 97% (*n* = 31) considered they have learned solid foundations about LA. This study shows face, content and construct validation and also educational value for this new low-cost, laparoscopic appendectomy model. The integration of this model in an introduction to laparoscopy course showed good results in regard to an increase of confidence among first-year surgery residents. It can be a valuable tool for learning and training laparoscopic appendectomy.

## Introduction

Appendicitis is a frequent condition worldwide and appendectomy has been considered the main treatment for both complicated and uncomplicated appendicitis [1]. Nowadays, minimally invasive appendectomy is being performed more frequently because of better results, such as reduced wound infection rates and hospital stays [2]. Thus, appendectomy simulation and training are becoming more and more important as this becomes the gold standard in most of the developed countries. Whether open or minimally invasive (MIS), appendectomy is one of the most frequent procedures performed by young residents. However, young residents find it difficult, especially on the division and ligation of the mesoappendix and the appendicular vessels [3]. In contrast to a few decades ago, nowadays, surgical education and training happen mostly out of the operating room [4]. Simulation allows trainees to gain experience in a safe environment using various types of models but available models are too simple and low fidelity or are very expensive and inaccessible for most of the trainees [5]. Like many other procedures, there is an increased need for appendectomy training opportunities, that would benefit if more models become available and accessible. Regarding laparoscopic appendectomy, cost-effective though realistic simulation models are not widely available and only a few training models exist [7, 8] and most of them not totally validated. To overcome these challenges we designed and tested a new and inexpensive model using different silicones, mimicking the anatomy, steps and pitfalls of LA. This study aimed to: (a) assess the fidelity, usefulness and educational value of the model; (b) assess the new methodology used in this introduction to laparoscopy course.

## Methods

The present study is a prospective cohort study based on procedural assessment and opinions/perspectives about a new low-cost laparoscopic appendectomy model and the methodology used in this curriculum.

### Participants

All the participants were first-year general surgery residents who started their residency on the 1st January 2024, in Portugal.

### Design

The hands-on session was carried out during an “Introduction to Laparoscopic Surgery Course,” on 20th April 2024, in Lisbon, Portugal. Course registration was free on a first-come, first-served basis, without active participant selection by the research team. First, participants were required to complete an online questionnaire (Google-forms®, Alphabet Inc., USA) regarding their demographic data and self-assessment of laparoscopic skills. All participants consented to participate in the course and in the study. Besides the simulation session with the model, the course was composed of other lectures and video sessions regarding MIS fundamentals. None of the residents had had a previous laparoscopic suture experience. Because of that, at the beginning of the course, there was a hands-on session of basic and intermediate dry-lab exercises and a masterclass on laparoscopic suture. As they were all first-year residents, and with no laparoscopic experience, we divided the group in two to differentiate them for the simulation and the test. Before the simulation session, both groups were instructed about the methodology and the assessment scale for the model. Additionally, Group A (Appendectomy video) was shown a video of the simulated procedure (Video—Supplementary file). After the course, they were asked to voluntarily and anonymously answer a post-course questionnaire about the course and the model, using five-point Likert scales.

### Appendectomy model

**Fig.1:**
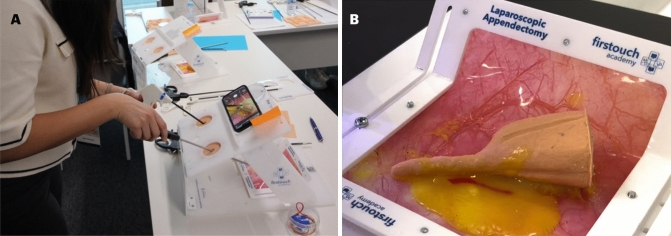
**A** Trainee with an individual training platform during the simulation; **B** Appendectomy model before use

A new appendectomy silicone model and the MIS First Trainer® platform (ACADEMIA FT, Portugal) were used for this course. Every trainee had one MIS platform (Fig. 1A) and one model (Fig. 1B). A panel of expert surgeons contributed to the development of the silicone model. This inexpensive model is made of silicone layers with a cost of approximately 60€. The structures represent the anatomy of the appendix and cecum, in their anatomical position. Mesoappendix fat and appendicular artery are also represented.

### Assessment

Along with the model production, a specific and objective assessment scale was created, with execution and quality scores, based on our previous experience [6].

### Steps of the Procedure

The model was placed into the MIS platform. Trainees used their own smartphone or tablet to perform the simulation, working both as MIS camera and MIS screen at the same time (Fig. 1A). A maryland and a clinch grasper were used to dissect the mesoappendix, isolate the appendicular artery and the appendix. Then Hem-o-lok® were used to ligate the appendix and the artery. After this step, both structures were sectioned with laparoscopic scissors and the rest of the appendix was isolated and appendectomy was carried out (Video—Supplementary file). During the simulation, trainees were instructed to pay special attention to precision, execution and quality (to avoid errors).

### Statistical analysis

All statistical analyses were performed using SPSS (IBM SPSS Statistics for Windows, Version 28.0). Descriptive data was presented as frequencies, means and standard deviation. T-Student tests were performed to compare means between the two independent groups.

## Results

### Participants

Thirty-five participants (*n* = 35), 63% females (*n* = 22) and 37% males (*n* = 13) were enrolled on the course and completed the simulation. The average age was 28 years (range 25–43, SD = 4,5). The group was divided into 2 sub-groups (A, *n* = 18 and B, *n* = 17). Both groups were presented real case videos and attended a lecture on how to perform a safe laparoscopic appendectomy. Additionally, group A was shown a video of a simulated appendectomy. All the participants concluded the course, meaning that they were able to perform all the basic and intermediate exercises and they were able to learn, practice and perform a complete laparoscopic appendectomy. Other characteristics of the participants are presented in Table 1.

**Table 1:** Characteristics of the participants

		*n*	%
Gender	Male	13	62,9
	Female	22	37,1
Age	25	9	25,7
	26	9	25,7
	27	8	22,9
	28	2	5,7
	29	1	2,9
	32	1	2,9
	34	1	2,9
	36	1	2,9
	38	1	2,9
	39	1	2,9
	43	1	2,9
Specialty	General surgery	35	100
Year of Residency	1st	35	100
MIS experience	None	35	100
Group	A (video)	18	51,4
	B (no video)	17	48,6

### Post-course questionnaire

The rate of response to the questionnaire was 91% (*n* = 32). The full results of the questionnaire can be found in Table 2.

**Table 2:** Model, methodology and course evaluation

Question/statement	Value	n	%	Mean	SD
How do you evaluate the Appendectomy session?	4—Good	10	31,3	4,69	0,471
	5—Excellent	22	68,8		
The model is adequate	4—Agree	14	43,8	4,56	0,504
	5—Totally Agree	18	56,3		
The model replicates the anatomy	4—Agree	16	50,0	4,5	0,58
	5—Totally Agree	16	50,0		
The model allows for laparoscopic appendectomy simulation	4—Agree	18	56,3	4,44	0,504
	5—Totally Agree	14	43,8		
You feel more confident after the simulation	4—Agree	14	43,8	4,56	0,504
	5—Totally Agree	18	56,3		
This methodology is adequate	4—Agree	11	34,4	4,66	0,483
	5—Totally Agree	21	65,6		
This methodology is more adequate than methodologies you found in other courses	3—Neither agree or disagree	1	3,1	4,44	0,564
	4—Agree	16	50		
	5—Totally Agree	15	46,9		
Watching a video of the procedure prior to the simulation is important	4—Agree	15	46,9	4,53	0,507
	5—Totally Agree	17	53,1		
How much impact will this course have in your laparoscopic skills?	4—High impact	12	37,5	4,63	0,492
	5—Very high impact	20	62,5		
How much impact will this course have in your Residency?	4—High impact	14	43,8	4,56	0,504
	5—Very high impact	18	56,3		
How much impact will this course have in your Career?	3—Neither high or low impact	2	6,3	4,38	0,609
	4—High impact	16	50		
	5—Very high impact	14	43,8		
You have learned solid knowledge about laparoscopic appendectomy	3—Neither agree or disagree	1	3,1	4,44	0,564
	4—Agree	16	50		
	5—Totally Agree	15	46,9		
How do you feel for your next laparoscopic appendectomy?	3—Neither more or less confident	1	3,1	4,44	0,564
	4—More confident	16	50		
	5—Much more confident	15	46,9		
How do you evaluate the course?	4—Good	11	34,4	4,66	0,483
	5—Excellent	21	65,6		
Would you recommend this course to your colleagues?	3—Maybe	1	3,1	4,59	0,56
	4—Yes	11	34,4		
	5—For sure	20	62,5		
What do you think about the course, regarding your expectations?	3—Neither higher or lower than expected	4	12,5	4,16	0,628
	4—Higher than expected	19	59,4		
	5—Much higher than expected	9	28,1		

### MIS simulation platform

The First Trainer® is part of the *Training4all Project* that we started in Portugal in 2017. In this project, every resident in Portugal gets one laparoscopic training platform with a set of instruments when they start surgical residency. It is a portable simulation platform to be used with a smartphone, a tablet or a laptop (with webcam). When asked about the First Trainer, the participants considered it as “important” or “very important” for their residency (51 and 36%, respectively) and “very adequate” for this kind of training (M = 4,72, *SD* = 0,457).

### Model evaluation

There was a strong agreement between participants for: model adequacy for this type of simulation/course (M = 4,56, *SD* = 0.504); face and content validation (M = 4,5, *SD* = 0,58; M = 4,44, *SD* = 0,58, respectively); and that this model gives more confidence to perform a real laparoscopic appendectomy (M = 4,56, *SD* = 0,504). Construct validation was also assessed (please see “3.6 Procedure assessment”).

### Methodology and course evaluation

Twenty-one participants (65%) totally agree that this new methodology was adequate for the course (M 4,66, *SD* = 0,483) and 96% “agree”/“totally agree” it is more adequate than other methodologies used in other courses (M = 4,44, *SD* = 0,564). Nearly 70% of the participants considered that the theoretical laparoscopic appendectomy session was “excellent” (M = 4,69, *SD* = 0,471). More than 95% of the trainees considered they had learned solid foundations about the procedure. Impact on residency, laparoscopic skills and career was considered “high”/“very high” (M = 4,56, SD = 0,504; M = 4,63, SD = 0,492; and M = 4,38, SD = 0,609, respectively). More than 87% considered that the course “exceeded”/“very much exceeded” their initial expectations. The course had a very good evaluation, with a mean of 4,66 (SD = 0,483) and 62,5% (*n* = 20) of the participants had no doubts in recommending the course to colleagues.

### Procedure assessment

The evaluation of the model after the course, applying the specific assessment scale, showed that global performance scores were higher in the group that had seen the pre-simulation video—group A (M = 12,82, SD = 2,21 vs M = 11,01, SD = 1,88, t(33) = 2,598, p = 0.007). Individual metrics for execution (M = 7,00, SD = 1,18 vs M = 6,05, SD = 1,34, t(33) = 2,197, *p* = 0.018) and quality (M = 5,82, SD = 1,41 vs M = 4,95, SD = 1,34, t(33) = 1,73, *p* = 0.046) were also higher in this group—Table 3

**Table 3:** Model assessment/performance

		*n*	Mean (M)	SD	*p*
Global Performance	Group A (video)	18	12,82 (out of 20)	2,21	0.007
	Group B (no video)	17	11,01 (out of 20)	1,88	
Execution	Group A (video)	18	7,00 (out of 10)	1,19	0.018
	Group B (no video)	17	6,05 (out of 10)	1,34	
Quality	Group A (video)	18	5,82 (out of 10)	1,42	0.046
	Group B (no video)	17	4,95 (out of 10)	1,55	

## Discussion

Simulation, disregarding its complexity, has proved to be fundamental to improve skills and this is especially true in the surgical and aviation fields [7]. It translates to the operating room, with an impact on performance, resulting in better clinical outcomes [8–11]. In previous research surveys we carried out, the need for more simulation and training opportunities was always pointed out as a high priority of young surgical residents. We also found that laparoscopic appendectomy is one of the earliest and most performed procedures among residents, including 1st year residents. Following those surveys, an “*introduction to MIS course”* was designed for first-year residents featuring theoretical knowledge and technical skills focusing on laparoscopic suture, laparoscopic appendectomy and laparoscopic cholecystectomy. Knowing the basics of MIS is mandatory to start simulating a MIS procedure so the course also covered fundamental aspects of MIS (ports, patient and team positioning; pneumoperitoneum; instruments and devices; among other topics).

Animal and cadaver models have proven their effectiveness and human cadavers have total fidelity regarding surgical simulation so we could have used these models instead. However, high cost, limited usage, ethical issues and special requirements represent challenges for these models to be widely adopted [12]. Virtual reality (VR) models were also an option but, at this time, are too expensive for democratizing simulation and creating training opportunities for residents [13]. On the other hand, silicone and dry-lab models are easier to store, transport, use and discharge. They can have less fidelity in comparison to cadaver and animal models but, usually, they can be used multiple times, without ethical issues and, in some cases, for a very low price. Silicone models proved to be easy to use, effective and allow for performance assessment.

In 2013, Omaira Rodriguez et al. described an inexpensive model for appendectomy. They used a latex glove with foam rubber taken from a surgical scrubbing brush for model production. Face and contact validation was not studied [6]. In 2017, Flemming Bjerrum et al. examined a virtual reality module for practicing a laparoscopic appendectomy as an assessment tool or training tool. There was no information about the cost for the simulation but it can be assumed as an expensive model as many VR simulators are. Like other VR models, it replicates real anatomy and structures but face and content validation weren’t assessed in this study. On the other side, the authors found that the differences between expertise groups (novices, intermediates and experienced) only occurred in some of the error parameters, thus concluding that procedural simulation may vary and future research is needed for better assessment [7]. Another model was published by Marina Yiasemidou et al. in 2020, using porcine large and small bowel, with a cost of 15 GBP. Forty-nine participants tested the model and construct validation was obtained using the Global Operative Assessment of Laparoscopic Skills (GOALS) score. Although the authors recommended the inclusion of this model to postgraduate surgical training, they only demonstrated construct validation but there were no results regarding face and content validation [8]. Later, in 2021, O’Connor and M Paraoan described an inexpensive model to simulate appendectomy using chicken wings. Before the simulation, they had to draw the human anatomy on the skin of the chicken to simulate the anatomical structures [9] and there was no information about the evaluation of the model. Recently, in 2023, Christopher W. Reynolds et al. described a new inexpensive model for Low and Middle-Income Countries (LMIC) using vinyl exam gloves as an appendix/cecum and a vessel loop or red yarn as an artery. Although the benefits for LMICs surgeons are obvious, face and content validations haven’t been assessed, probably because those materials cannot replicate the real anatomy, aspect, dimensions and texture of the real structures. Given these examples, one can conclude that creating a triple-validated and inexpensive appendectomy model is not an easy task. Regarding laparoscopic appendectomy, validated simulation models are not widely available [14, 15]. Our team has been working with silicone and low-cost models for more than 5 years [6, 16] so, as in other previous courses, we decided to design a specific silicone model for laparoscopic appendectomy simulation. A panel of expert surgeons contributed to the design and assessed the model before its final production. This is a low-cost model, produced with small silicone parts and layers placed to mimic human anatomy and feel. Each training opportunity costs nearly 60€ and there is no need for specific facilities or heavy logistic. As in real-life appendectomy, the appendix and artery must be isolated, ligated and sectioned. This model replicates the most important structures regarding a laparoscopic appendectomy: the appendix, the mesoappendix, the cecum and the appendicular artery, and all these structures are laying down, on top of the “retroperitoneum” and medial to the right paracolic gutter to give a sense of being inside an abdominal cavity (Fig. 1B)**.**

Interestingly, during the session, we observed that this was the first time some of the residents were using real laparoscopic instruments. Additionally, it was the first time for all of them to use ligation clips. This proves that this kind of course and models are essential for young resident’s training, as they are given the opportunity to handle devices and instruments before using them on real patients.

**Fig. 2:**
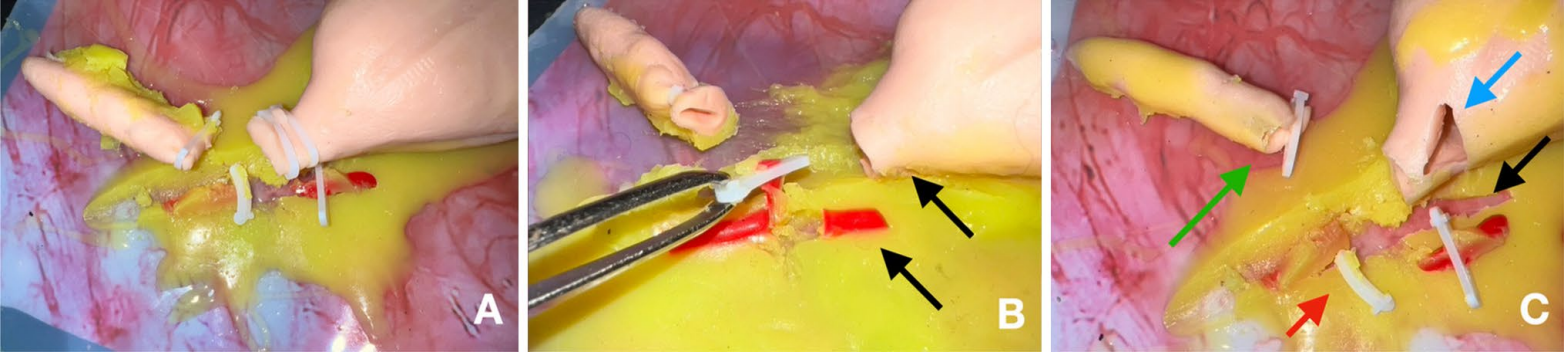
Models after the simulation procedure. **A** No errors; **B** black arrows—absence of proximal clip on the artery (minor error) and absence of appendix proximal clips (major errors); **C** green arrow—partially lost distal clip on appendix (minor error); blue arrow—laceration of the cecum (major error); red arrow—loose distal clip on artery (minor error); black arrow—artery ligated but ripped out of its bed (major error)

After the simulation session, the models were photographed and assessed using the specific scale of the model (Fig. 2). Simulation assessment is essential for good learning outcomes, to understand the steps of the operation and avoiding errors. One of the advantages of in-house design of the model is that we had the capability of adding features and characteristics that allowed us to construct an objective assessment scale for the model. In our scale, trainees start with 0 “Execution points” and 10 “Quality points” and points are summed or rested depending on the performance. “Execution” points refer to the completion of procedural steps and “Quality" points refer to errors during the execution (Fig. 2). These errors are classified differently, depending on the negative outcome they represent for the patient (starting in "low quality” > “minor error” > “major error” > “critical error”) and a different amount of points is attributed to each type of error. To illustrate this, as an example, “low quality” refers to an appendix not skeletonized (− 0,3 points). A lost clip can be considered as a “minor error” (− 0,8 points) as the trainee can correct it and place another one. A “major error” (− 1,2 points) would occur if the exercise is finished without clipping the appendix or the artery. Although they could be surgically corrected they would represent a bad outcome for the patient. Finally, a “critical error” (− 1,5 points) would be a situation that is not usually expected or not easily solved, needing: conversion to open surgery; referral to dedicated centers; additional or emergency surgical procedures; or would represent high morbi-mortality to the patient (e.g. damage or lesion to the cecum). This categorization of errors highlights the importance of different complications in the surgical outcome. This scale allows for an objective assessment and also helps trainees to understand how to improve through continuous practice. To our knowledge, there is no laparoscopic appendectomy simulator that has a specific objective assessment scale adapted to it. We consider this is very important as other validated scales are non-model specific or lack some objectivity. In addition, our results showed that the model was well received by the participants and allowed for face, content and construct validation of the model.

A majority of participants highly evaluated the course, the sessions, the faculty and the methods used. Most of them agreed that this methodology is better than others and it should be used in the other courses of our training program. We think that it is really important that trainees agree that the course will have a considerable impact on their laparoscopic skills, their residency and career. Most important, though, is that they feel more confident to perform their first appendectomy on real patients.

## Conclusions

This pilot study provided evidence for the use of a novel methodology, a new low-cost simulation model and a specific scale for appendectomy simulation and assessment. It showed face, content and construct validation for this new silicone model. These findings show that, although low-cost and simple, this model allows for useful simulation, learning and skills assessment. We think it can be a valuable tool for learning and training laparoscopic appendectomy, which is one of the first procedures that general surgery residents perform at the beginning of residency. This, combined with the possibility of having a portable MIS platform at home and easily accessible models, is key to allow for continuous practice during residency. The integration of this low-cost model in this new methodology showed good results in regard to an increase of confidence among first-year surgery residents. Additionally, this work also supports that introduction to MIS courses is well received by residents at the early stages of their career, with a perceived impact on their laparoscopic skills, residency and career.

## Sources of funding for research and/or publication:

This manuscript did not receive any specific grant from funding agencies in the public, commercial, or not-for-profit sectors.

## Data Availability

All data access is granted upon request to the authors.
